# Clinicopathological features of breast cancer in Ukrainian women with BRCA1 c.181T>G (p.Cys61Gly) variant: a case series

**DOI:** 10.3389/fendo.2026.1859939

**Published:** 2026-06-30

**Authors:** Nataliya Kitsera, Yaroslav Shparyk, Orest Tril, Ihor Protsko, Ivanna Duda, Olha Oleksyak, Andrii Hrynkiv, Irena Dovhanyk, Iryna Hrytsai, Iryna Tril, Halyna Makukh, Alina Kruhlykova, Denys Kozakov, Sofiia Livshun, Nazarii Kobyliak, Solomiia Sobechko, Myroslava Oliyarnyk, Oksana Sulaieva

**Affiliations:** 1 Lviv Oncologic Regional Treatment and Diagnostic Center, Lviv, Ukraine; 2 Ivano-Frankivsk National Medical University, Ivano-Frankivsk, Ukraine; 3 Scientific and Medical Genetic Center “LeoGENE”, Lviv, Ukraine; 4 Medical laboratory CSD, Kyiv, Ukraine; 5 Ukrainian Association for Precision Medicine, Kyiv, Ukraine; 6 Institute of Molecular Biology and Genetics National Academy of Sciences (NAS) of Ukraine, Kyiv, Ukraine; 7 Bogomolets National Medical University, Kyiv, Ukraine

**Keywords:** BRCA1, breast cancer, c.181T>G variant, family history, germline variant, tumor biology

## Abstract

**Introduction:**

*BRCA1* c.181T>G (p.Cys61Gly) is a pathogenic founder genetic variant prevalent in Central and Eastern Europe. The data on its clinicopathological manifestations in Ukrainian patients remain limited.

**Aim:**

This study aimed to characterize clinical presentation, tumor biology, and family history in Ukrainian women with BRCA1 c.181T>G variant.

**Materials and methods:**

We conducted a single-centre case series of women with primary breast cancer (BC) and/or ovarian cancer (OC) harbouring *BRCA1* c.181T>G genetic variant and treated at Lviv Regional Oncology Treatment and Diagnostic Center (Ukraine) between January 2024 and August 2025. Clinical presentation, tumor pathology, biomarker status, family history, treatment, and outcomes were analysed.

**Results:**

Thirteen women aged 29–81 years (median 35 years) were included in this case series. Early-onset BC was recorded in 10 (76.9%) of patients diagnosed before 40 years. BC was the initial malignancy in 12 (92.3%) patients. OC occurred as a first tumor in 1 (7.7%) and as a subsequent cancer in 4 (30.8%) patients. Multiple malignancies were observed in 61.5% of patients, with intervals of 5–21 years between diagnoses. Family history revealed strong clustering of BC and OC across generations, often involving multiple affected relatives, consistent with hereditary breast and ovarian cancer syndrome phenotype. Among cases with known biomarker status (10/13), 2 were HER2-positive, 4 belonged to luminal-like type, and 4 cases represented triple negative BC. Most cases (10/13, 76.9%) were diagnosed at early tumor growth stage (pT1-2). However, more than half of primary tumors (8 of 13; 61.5%) had positive nodal status (pN1-2) reflecting invasive behaviour of cancer cells.

**Conclusions:**

This Ukrainian case series demonstrates that the *BRCA1* c.181T>G genetic variant is associated with early-onset breast cancer, variable tumor biology, frequent multiple primary cancers, and strong familial clustering. These observations support the need for expanded genetic testing, targeted surveillance, and corresponding risk reduction strategies tailored to Ukraine’s *BRCA1* founder pathogenic variant landscape.

## Introduction

It is well established that *BRCA1* plays a pivotal role in maintaining genomic stability and tumor suppression. Specific point pathogenic variants in this gene are consistently associated with elevated susceptibility to breast and ovarian cancers (1–3). The c.181T>G (p.Cys61Gly) pathogenic variant (also known as 300T>G or C61G) maps to exon 5 of *BRCA1* and alters an amino acid residue in the RING domain of the corresponding protein. This sequence change replaces the highly conserved cysteine at codon 61 with glycine, disrupting BRCA1 protein function, impairing DNA repair and promoting oncogenesis (4).

The c.181T>G variant is recognized as a founder pathogenic variant in several Central and Eastern European populations (5, 6). In Poland, Romania and the Czech Republic it ranks second or third most frequent *BRCA1* pathogenic genetic variant (7–11). This pathogenic variant belongs in the top three founder pathogenic variants in women of Ashkenazi Jewish ancestry (12). A recent study reported high prevalence of *BRCA1* c.181T>G genetic variant among Ukrainian women with breast cancer (13). At the same time, in Italy, this pathogenic variant is less common compared to other BRCA1 genetic variants associated with breast cancer (14). It was found in 1.3% breast cancer women accounting for 8.4% of *BRCA1/2* carriers.

Molecular and epidemiological evidence confirms the pathogenicity and disease association of *BRCA1* c.181T>G variant. This is supported by functional evidence demonstrating impairment of structural integrity and zinc-binding capacity of *BRCA1* RING domain. This disrupts *BRCA1* tumor suppressor function. Although the number of large-scale population-specific studies of *BRCA1* variants in Ukraine remain limited, available data indicate that Ukrainian patients have both common founder and rare *BRCA1/2* genetic variants (15). Moreover, the spectrum of clinicopathological features in Ukrainian patients with breast cancer is still unclear.

This study aimed to evaluate family history, clinical presentation and tumor biology in Ukrainian women with germline *BRCA1* c.181T>G genetic variant.

## Materials and methods

This case series study included women with breast cancer (BC) who received care at Lviv Regional Oncologic Treatment and Diagnostic Center (Ukraine) between January 2024 and August 2025. The study cohort represents all consecutive *BRCA1* c.181T>G carriers identified during the study period who underwent genetic counseling. No patients were excluded. Written informed consent was obtained from all participants for genetic testing and the use of their clinical and genetic data for research purposes. All patients underwent germline *BRCA1/2* testing. Clinical, genetic, and demographic data were retrieved from medical records. Patients with BC and BC/OC combinations with *BRCA1* c.181T>G variant were included in this study. Each patient underwent genetic counselling, and family cancer history was collected.

Genetic testing was performed at CSD LAB (Kyiv, Ukraine) via next generation sequencing (NGS) and in LeoGene laboratory (Lviv, Ukraine) via PCR. 9 patients were tested by NGS, and 4 patients underwent PCR testing for germline *BRCA1/2* variants, confirmatory testing was not performed. Peripheral blood samples were collected from patients into K2 EDTA tubes. Genomic DNA was extracted from leukocytes using the E.Z.N.A.^®^ Blood DNA Mini Kit (Omega Bio-tek, USA). DNA quantification was performed using a spectrofluorimetric method (DeNovix dsDNA Broad Range Assay on a DeNovix QFX fluorometer; DeNovix, USA). NGS library preparation was performed using the BRCA Pro Panel kit (AmoyDx, China) according to manufacturer’s instructions. Sequencing was performed on a NextSeq 550Dx platform (Illumina, USA), and NGS data were analysed using the ANDAS ADXHS-gBRCA v1.5.0 server (AmoyDx, China). Data analysis workflow included quality control of FASTQ files, alignment of sequencing reads to the human reference genome (GRCh37), variant calling, and functional annotation. Detection of *BRCA1 c.181T>G* variant by real-time PCR was conducted using GeneMap BRCA1&2 Founder Pathogenic Variants Detection Kit (Turkey). Amplification and signal detection was performed on a Bio-Rad CFX96 real-time PCR system (USA) according to the manufacturer’s instructions. Data were analysed using «CFX Maestro» software with automatic Ct determination and quality control. The presence of mutant allele was evaluated based on target-specific threshold values.

## Results

Thirteen women aged 29 to 81 (median – 35 years; IQR 32.8-40.8 years) with BC carrying *BRCA1* c.181T>G genetic variant, treated at Lviv Regional Oncologic Treatment and Diagnostic Center (Ukraine), underwent genetic counselling between January 2024 and August 2025. Patient clinical features, tumor pathology and biomarker status are presented in **Table 1**.

**Table 1:** Clinicopathological characteristics of study participants.

Characteristics	Number	%
Age of BC onset		
under 40	10	76.9%
over 40	3	23.1%
Age of BC onset		
under 50	12	92.3%
over 50	1	7.7%
Laterality of BC		
Bilateral BC	3	23.1%
Unilateral BC	10	76.9%
Combination with OC		
Metachronous OC	5	38.5%
Primary OC	1	7.7%
No OC	7	53.8%
Staging of primary tumor		
T1-2	10	76.9%
T3-4	3	23.1%
N0	5	38.5%
N1-2	8	61.5%
M0	13	100%
M1	0	0%
Molecular characteristics		
HR+	6	46.2%
HR-	4	30.8%
ND	3	23.1%
HER2-positive	2	15.4%
HER2-negative	8	61.5%
ND	3	23.1%
Disease course		
Disease progression	3	23.1%
Remission	10	76.9%

*ND – cases with no data available concerning IHC.

The age of cancer onset ranged widely from 29 to 74 years. In 10 (76.9%) of 13 patients, BC was diagnosed before the age of 40, and in 12 of 13 cases (92,3%) – before 50 (**Table 1**). In 12 of 13 probands (92,3%) BC was the primary malignancy. In one case, ovarian cancer (OC) was the primary diagnosis followed by later BC onset. Majority (8 of 13; 61.5%) of women in the study had metachronous BC or OC. Among them, 5 (38.5%) patients possessed BC with metachronous OC, 2 had bilateral metachronous BC and one was diagnosed with bilateral BC and metachronous OC. Time to second malignancy onset was over 10 years in all cases. The shortest latency period was 5 years (patient №6, contralateral breast cancer), while the longest one was 21 years (patient №2, metachronous ovarian cancer).

Tumor staging revealed variability of tumor characteristics. Most cases (10/13, 76.9%) were diagnosed at early tumor growth stage (pT1-2). However, more than half of primary tumors (8 of 13; 61.5%) had positive nodal status (pN1-2) reflecting invasive behaviour of cancer cells. Among cases with known HR status (10/13) 6 (46.2%) were hormone-sensitive and 4 (30.8%) demonstrated the loss of HR expression.

**Figure 1:**
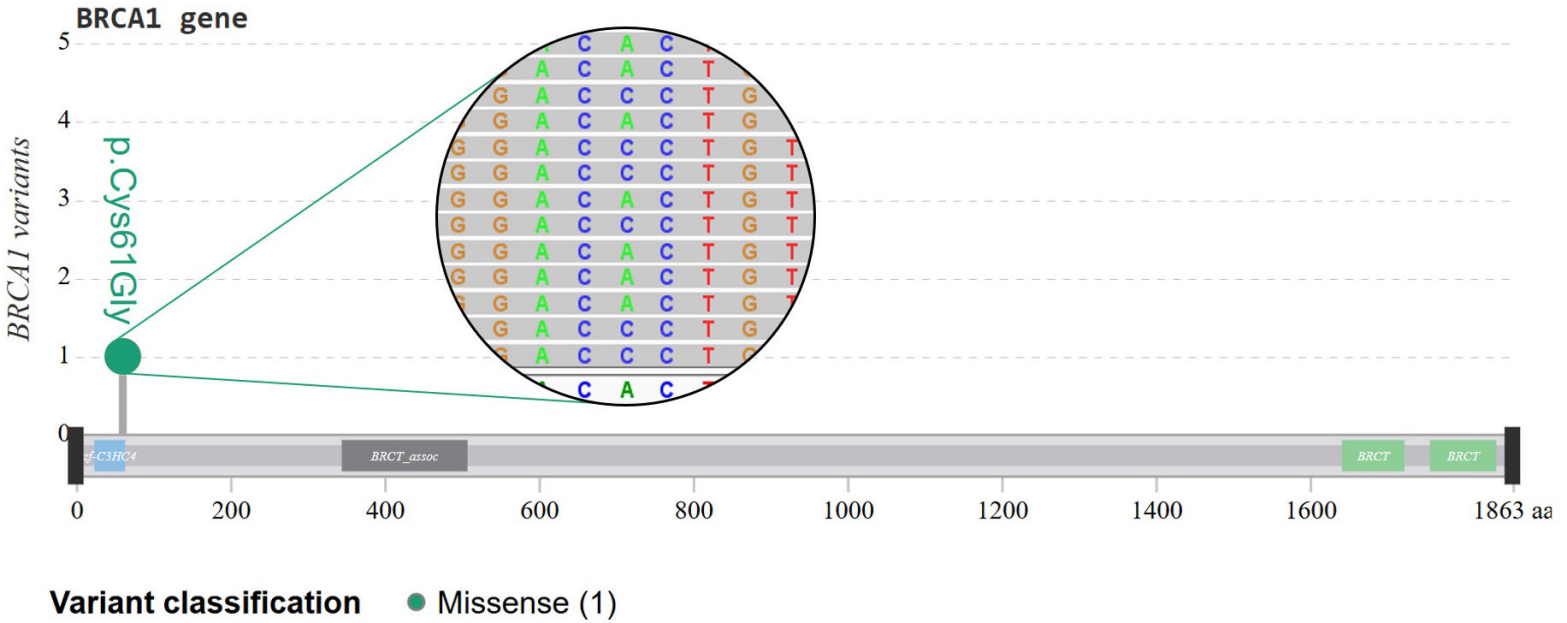
Schematic representation of BRCA1 c.181T>G genetic variant. The diagram illustrates the structural organization of the BRCA1 gene and its functional domains, highlighting the localization of the c.181T>G transversion in exon 5. This pathogenic nucleotide substitution results in a missense transformation at the protein level (p.Cys61Gly), disrupting the highly conserved zinc-binding residues within the N-terminal RING finger domain (amino acids 24–108), which is critical for BARD1 interaction and ubiquitin ligase activity.

HER2 status was predominantly negative across the dataset. Only 2 (15.4%) tumors exhibited HER2 positivity, and both were HR-positive. Therefore, among 10 tumors with known biomarker status, 2 were Her2-positive, 4 belonged to luminal-like subtype, and 4 represented triple negative BC (TNBC) (**Figure 1**).

Family cancer history of 13 women with *BRCA1* c.181T>G genetic variant is summarized in **Table 2**. Across the cohort, most probands reported multiple cases of cancer among first-, second-, or third-degree relatives, indicating the presence of hereditary cancer predisposition, associated with the observed BRCA1 variant. The number of relatives with cancer varied among patients, ranging from 1 to 7 affected family members. Notably, families with multiple affected members often showed vertical inheritance of breast and ovarian cancers across two or more generations. BC was the most frequent diagnosis, occurring in all 13 families (100%), often at young ages (30s–40s). OC was documented in 5 families (38.5%). In several families, clustering of two or more BC/OC cases across generations was observed, indicating strong hereditary patterns.

**Table 2:** The family cancer burden in women with breast or breast/ovarian cancer and *BRCA1 c.181T>G* pathogenic variant.

Family №	№ of relatives with cancer	Cancer types reported in relatives	Healthy BRCA1 c.181T>G carriers	Healthy non-carriers
1	1	Leukaemia (n=1)	Sister (44), Father (74)	Mother (74)
2	1	BC (n=1)	–	–
3	2	OC (n=1), BC (n=1)	–	–
4	3	BC (n=2), Gastric cancer (n=1)	Sister (43)	–
5	3	BC (n=1), OC (n=1), Leukaemia (n=1)	–	–
6	1	BC (n=1)	–	–
7	7	BC (n=4), OC (n=1), Pancreatic cancer (n=1), Lung cancer (n=1)	–	–
8	2	OC (n=1), Lung cancer (n=1)	–	–
9	2	BC (n=1), Leukaemia (n=1)	–	–
10	1	Liver cancer (n=1)	Father (68)	Mother (66)
11	5	BC (n=5)*	Maternal aunt (48)	Sister (42), Cousin (26)
12	2	OC (n=1), Prostate cancer (n=1)	Son (30), Daughter (28)	–
13	2	BC (n=1), Unspecified cancer (n=1)	–	–

*Including one male of BC case.

**Figure 2:**
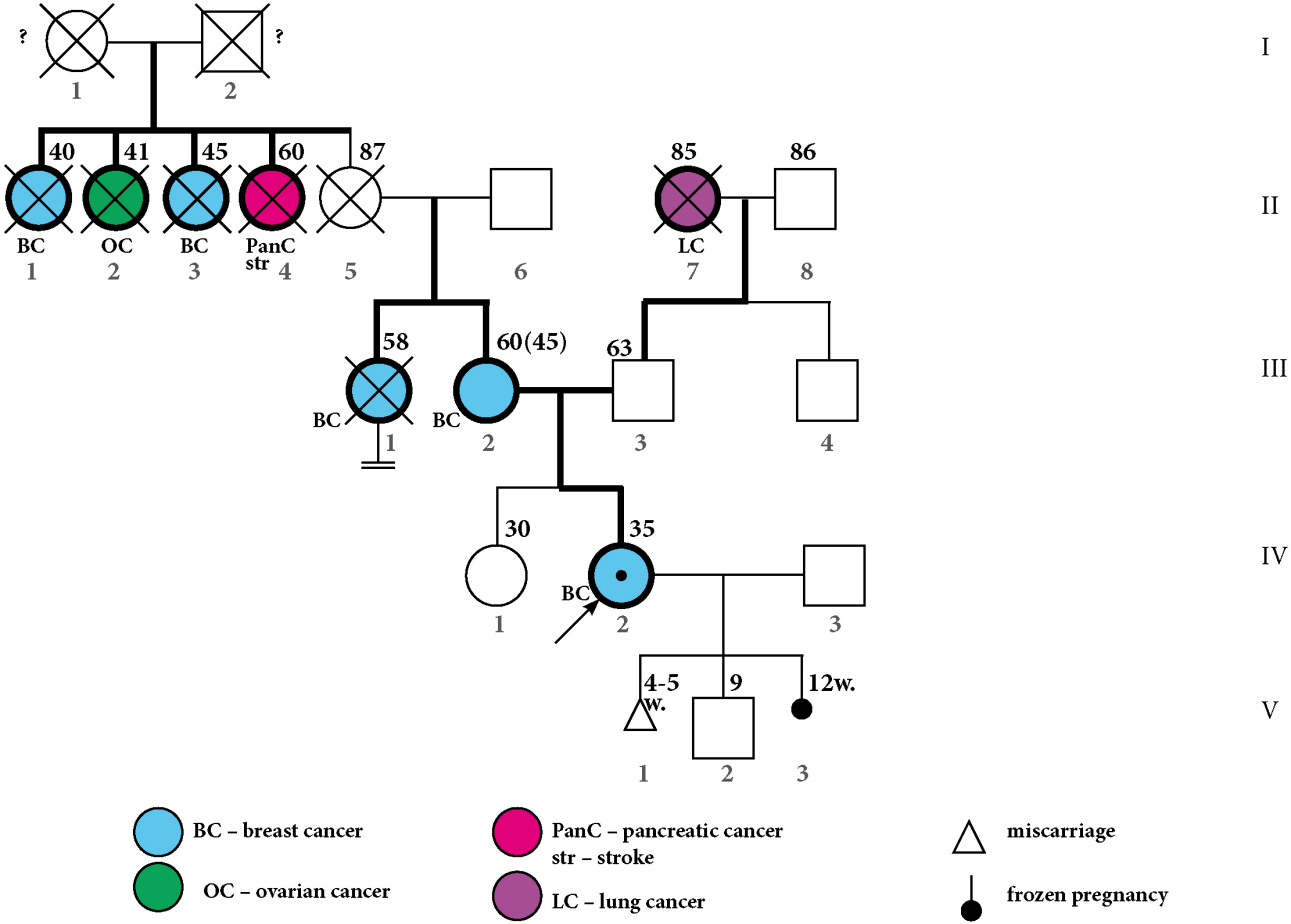
Family tree of patient №7 with BRCA1 c.181T>G and history of cancer. The pedigree illustrates an inheritance pattern highly consistent with an autosomal dominant hereditary breast and ovarian cancer (HBOC) syndrome. Squares represent males, circles represent females, diagonal slashes indicate deceased individuals. Arrow identifies the proband (IV-2), who was diagnosed with early-onset BC at age 35 and carries the pathogenic BRCA1 c.181T>G variant. Affected maternal relatives in generation II had breast, ovarian, and pancreatic malignancies. Generations III and IV track early-onset BC segregation, while generation V displays individual pregnancy losses without oncologic manifestations to date.

Several pedigrees also included OC, prostate cancer, pancreatic cancer, and other malignancies. The pedigree of patient №7 revealed a dense clustering of malignancies, with numerous aunts and grandparents affected by breast, ovarian, and pancreatic cancers (**Figure 2**). In the II^nd^ generation on the maternal side 4 siblings were affected: two with BC (II-1, II-3), one with OC (II-2), and one with pancreatic cancer (II-4). In the II^nd^ generation, there was also a case of lung cancer on the paternal side (II-7). The III^rd^ generation demonstrated early-onset breast cancer in two women. One of the sisters developed BC at 45 years (III-2) and the elder sister (III-1) died of BC at 58 and had a childless marriage. In the IV^th^ generation, the elder daughter (IV-2) of III-2 was diagnosed with BC at age 35, while her younger sister (IV-1) remained unaffected. The V^th^ generation encountered pregnancy losses (V-1, V-3), but no cancer diagnoses to date. Overall, the pattern suggests a hereditary cancer syndrome, consistent with a BRCA1/2-associated phenotype. The proband (IV-2) was found to have *BRCA1* c.181T>G pathogenic genetic variant.

This analysis highlights that the *BRCA1* c.181T>G variant is associated with extensive family histories of cancer, with strong clustering of breast and ovarian cancers, but also with occurrence of other malignancies. In addition to cancer diagnoses, some pedigrees reported non-cancer conditions (e.g., congenital heart defects, Turner syndrome, developmental delay, cystic kidney/liver disease, goitre), which may reflect broader family health characteristics. The observed clinical characteristics of 13 patients with BC and BC/OC, including disease course, treatment tactics and long-term outcomes are represented in **Table 3**.

**Table 3:** Features of treatment in women with breast or breast/ovarian cancer and *BRCA1 c.181T>G* pathogenic variant.

Patient	Age at first cancer diagnosis (years	Cancer history	Bilateral BC	Ovarian cancer	Disease progression/recurrence	Current status (September 2025)
1	29/29	BC/OC	No	Yes	Bone and brain metastases (12 years)	Alive
2	30/51	BC/OC	No	Yes	No	Alive
3	32/48	BC/OC	No	Yes	Ovarian recurrence (1 year)	Alive
4	33/47	Bilateral BC	Yes	No	No	Alive, 10 years
5	34/44/54	BC/OC/contralateral BC	Yes	Yes	Ovarian and retroperitoneal lymph node recurrence (3 years)	Alive
6	34/39	Bilateral BC	Yes	No	No	Alive
7	35	BC	No	No	No	Alive
8	37	BC	No	No	Not yet assessable	Alive
9	38	BC	No	No	No	Alive
10	40	BC	No	No	No	Alive
11	43	BC	No	No	No	Alive
12	49/58	BC/OC	No	Yes	No	Alive
13	74/81	OC/BC	No	Yes	No	Alive

Neoadjuvant chemotherapy was administered in 7 (53.8%) cases, mainly in more aggressive or advanced forms. Surgical treatment included mastectomy, quadrantectomy and bilateral mastectomy for BC, as well as panhysterectomy and oophorectomy for ovarian tumors. Most patients underwent breast-conserving or combined surgery, while reconstructive interventions using implants were used in only 2 (15.4%) cases. Adjuvant chemotherapy and hormonal therapy were administered in 9 (69.2%) and 3 (23.1%) cases, respectively, reflecting the different biomarker status of tumors. Targeted therapy and PARP inhibitors were used to a limited extent, which may be related to the clinicopathological characteristics of the tumor or the affordability of the medication. Disease progression was recorded in 3 (23.1%) patients with metastatic lesions in the bone, brain or ovaries. These women underwent repeated chemotherapy treatment. The duration of follow-up ranged from 1–10 years; however, the small sample size precludes conclusions regarding prognosis.

## Discussion

The clinicopathological profile observed in our cohort is consistent with previously reported *BRCA1*-associated populations from Central and Eastern Europe. The *BRCA1* c.181T>G (p.Cys61Gly) variant is recognized as a high-risk pathogenic founder variant in this region (5, 8). Large studies of *BRCA1* pathogenic variant carriers have demonstrated early disease onset, increased risks of bilateral breast cancer and ovarian cancer. In our cohort, 76.9% of patients were diagnosed before the age of 40, 23.1% developed bilateral breast cancer, and 38.5% experienced metachronous ovarian cancer, supporting the similarity of Ukrainian *BRCA1* c.181T>G carriers to other founder pathogenic variant populations. Although several tumors exhibited features commonly associated with *BRCA1*-related breast cancer, including high histological grade, hormone receptor negativity, and elevated Ki-67 expression, the prevalence of triple-negative breast cancer could not be reliably assessed because receptor status data were unavailable for several patients (16).

In our cohort, the median age of first cancer manifestation was notably young, with cancers occurring as early as 29 years old. This aligns with European registry data, where patients with c.181T>G are often diagnosed in their 30s or 40s, much earlier than sporadic cases (7). Consistent with recent data, 4 of 10 tumors with known HR status tumors in our study displayed TNBC characteristics, reflecting the molecular consequences of *BRCA1* dysfunction in homologous recombination repair (17). At the same time 2 of 10 BC with available receptor status data were HER2 positive and 4 belonged to luminal-like subtype, reflecting heterogeneity of tumor biology in carriers of *BRCA1* c.181T>G (p.Cys61Gly) variant. Elevated Ki-67 levels in our patients reinforce the notion of an aggressive proliferative biology, which has been linked to poorer prognosis and a greater likelihood of recurrence in *BRCA1*-driven tumors (18).

The geographical distribution of *BRCA1*c.181T>G demonstrates its relevance as a regional founder pathogenic variant. In Poland, Czech Republic, and Romania, it remains among the top three most frequent pathogenic variants (8, 11, 19). Our data support this pattern to Ukraine, where population-wide genetic epidemiology studies are still limited (15, 20).

Our genealogical analysis revealed extensive clustering of breast and ovarian cancers across maternal and paternal lineages, often spanning multiple generations. The family histories of the 13 women carrying *BRCA1* c.181T>G genetic variant demonstrate patterns consistent with hereditary breast and ovarian cancer (HBOC) syndrome. The prevalence of BC among first- and second-degree relatives aligns with previous observations that *BRCA1* pathogenic variants strongly predispose carriers and their close relatives to early-onset BC (21) and ovarian cancer (22, 23). The wide range in the number of affected relatives, spanning from 1 to 7, illustrates the heterogeneity of familial cancer burden among *BRCA1* families, despite high penetrance (24, 25). These findings underscore the importance of detailed pedigree analysis for identifying individuals at increased hereditary cancer risk and guiding genetic testing strategies. In addition to breast and ovarian cancers, several non-HBOC malignancies, including gastric cancer, liver cancer, and leukaemia, were reported in the family histories of some carriers (26). However, the relationship between *BRCA1* pathogenic variants and these malignancies remains uncertain, and current evidence does not support their inclusion within the established *BRCA1*-associated cancer spectrum. Therefore, these observations should be considered hypothesis-generating and interpreted with caution.

The presence of family history of cancers not associated with HBOC, including gastric and hematologic cancers, aligns with the emerging evidence that *BRCA1* pathogenic variants may confer moderate risks for several non-HBOC tumors (27). The detection of genetic variants predominantly by NGS reflects the recent diagnostic shift toward comprehensive genetic testing in Ukraine, which improves identification of both common and rare *BRCA1* variants, as was shown by Lerner-Ellis J (28).

The limitation of this study relied on case series design with observational character of the data and variable follow up periods. In addition, the incomplete availability of immunohistochemical data, particularly hormone receptor and HER2 status, for several patients. This restricted the assessment of molecular subtypes and may have influenced estimates of the prevalence of triple-negative breast cancer within the cohort.

## Conclusion

This Ukrainian case series showed that patients with *BRCA1* c.181T>G pathogenic genetic variant predominantly develop early-onset breast cancer, mostly occurring before 40 years, demonstrating strong familial clustering. Metachronous ovarian cancer and bilateral or metachronous breast cancers were common, often arised more than a decade after the first diagnosis, indicating persistent lifelong cancer risk. Despite biological heterogeneity, most patients achieved remission following multimodal treatment, underscoring the importance of genetic counselling and long-term surveillance for women carrying this high-risk *BRCA1* founder variant.

## Data Availability

The raw data supporting the conclusions of this article will be made available by the authors, without undue reservation.
